# A patient-derived benchmark for evaluating large language models in connective tissue diseases: blinded multi-stakeholder assessment and guideline comparison

**DOI:** 10.1007/s00296-026-06178-1

**Published:** 2026-07-14

**Authors:** Phillip Kremer, Johannes Knitza, Anna Reichard, Marie Therese Holzer, Tingting Xiong, Ulrike Schnoor, Simon Melderis, Ina Kötter, Isabell Haase, Martin Krusche

**Affiliations:** 1 III. Department of Medicine, University Medical Center Hamburg-Eppendorf, Hamburg, Germany; 2 Institute for Digital Medicine, Philipps-Universität Marburg, University Hospital of Giessen and Marburg, Baldingerstrasse, Marburg, Germany; 3 Rheumazentrum Lübeck, Lübeck, Germany; 4 Clinic for Rheumatology and Clinical Immunology, Auenlandklinik Bad Bramstedt, Bad Bramstedt, Germany

**Keywords:** Artificial intelligence, Digital health, Patient care, Patient questions, Myositis, Systemic sclerosis, Systemic lupus erythematosus, Sjögren disease, Large language models, Consumer health information, Patienteducation as topic, Connective tissue diseases, Scleroderma, Sjogren's syndrome

## Abstract

To co-develop disease-specific patient questions for connective tissue diseases (CTDs), compare patient/rheumatologist ratings of answers from language models (LLMs) versus Google Search, and quantify EULAR coverage of these patient-prioritized questions. In this prospective single-center observational study, reported in accordance with STROBE, patient advocacy groups curated 20 frequently asked questions (FAQs) for each CTD (systemic lupus erythematosus, idiopathic inflammatory myopathy, Sjögren disease, systemic sclerosis). Questions were submitted to Claude 4.0 Sonnet, ChatGPT-5, Gemini 2.5 Pro, and Google Search. Five patients per disease and five rheumatologists rated blinded outputs using forced-rank preference and 5-point Likert scales (patients: empathy, trustworthiness, comprehensibility; physicians: medical correctness, empathy, comprehensibility). Guideline mapping assessed whether EULAR recommendations addressed each question. All three LLMs answered 100% of questions (80/80), whereas Google Search answered 90% (72/80). Across CTDs, patients rated LLM outputs favorably for empathy (mean 1.40–2.77), trustworthiness (1.47–2.6), and comprehensibility (1.33–2.27), and rheumatologists for medical correctness (1.23–1.98). Patients and physicians most often ranked Gemini 2.5 Pro best overall (rank 1: 59% and 63%), and Google Search most often worst (rank 4: 55% and 57%). Cluster analyses showed no consistent differences across five clusters. Guideline mapping revealed substantial gaps: 40–55% of prioritized FAQs were not addressed by EULAR recommendations, with largest gaps in cluster E, life impact, psychosocial aspects and family planning (75–100% not addressed; 0% fully addressed). In this blinded evaluation, LLMs produced CTD FAQs responses patients perceived as empathic, trustworthy and rheumatologists rated medically correct, with Gemini 2.5 Pro most consistently preferred overall. However, the mismatch between prioritized questions and guideline coverage underscores the need for patient-centered, evidence-grounded information resources.

**Supplementary Information:**

The online version contains supplementary material available at 10.1007/s00296-026-06178-1.

## Introduction

Rheumatic and musculoskeletal diseases (RMDs) are among the most common chronic non-communicable diseases [1]. They contribute substantially to the overall societal burden of functional limitation and place significant demands on healthcare and social care systems [2]. Patients with connective tissue diseases (CTDs) constitute a particularly relevant subgroup in rheumatology due to the complex, multisystem conditions, variable disease courses, and the resulting substantial need for reliable, disease-specific guidance [3]. Reflecting this, current EULAR guidelines consistently emphasize the importance of patient education as a core component of disease management [4, 5]. In patients with RMDs, disease-related knowledge and health literacy (HL) are directly interlinked with favorable and equitable health outcomes [6]. Therefore, providing clear and comprehensive information about the disease may help improve HL for patients with RMDs [7–9].

Given the worsening shortage of rheumatologists [10], patients are increasingly using alternative paths for information gathering, such as searching the internet for health-related information [11]. Therefore, in recent years, internet search engines such as Google have increasingly been consulted for medical inquiries. While reliance on the internet in this context is growing, with 42% to 71% of patients primarily accessing online platforms for information, searching for and evaluating online content poses real challenges for many patients [9, 11–13]. As a result, the term “Dr. Google” has emerged, underscoring the growing role of this search engine as an initial source of health information. However, this trend has also facilitated the spread of misunderstandings and medical misinformation due to wide variability in the quality of online content and patients’ limited ability to critically appraise such information [14].

With the rapid emergence of large language models (LLMs) over the past years, online search and information-seeking behavior has further evolved. Early pilot studies suggest that these systems are increasingly used by patients [15] and may offer substantial benefits in addressing medical questions and supporting patients in navigating health information [16–18]. Recent studies in rheumatology have evaluated LLMs for patient-related information, diagnostic reasoning, treatment decision support, and rheumatology benchmark questions, reporting promising but heterogeneous results and emphasizing the need for expert oversight and standardized evaluation frameworks [15, 19–22]. For instance in familial Mediterranean fever LLMs generally achieved high but model-dependent accuracy when answering patient-focused questions, with occasional inconsistencies and misinformation [23]. Beyond this, Forte et al. showed that ChatGPT-4 generated accurate and readable responses to patient-prioritized questions and was often preferred by patients over rheumatologist-written answers, highlighting the communication quality in patient-facing LLM applications [24].

Despite their considerable potential, LLMs have not yet been systematically assessed in CTDs regarding the accuracy and trustworthiness of their responses to real-world patient questions, nor directly compared with traditional Google-based searches. Therefore, this study aimed to (1) develop consensus-based, disease-specific lists of the most important patient questions; (2) evaluate patient and physician perceptions of LLM–generated answers to these questions; and (3) examine whether the patient-derived benchmark questions are addressed in current disease EULAR guidelines.

## Methods

This prospective, single-center study compared the quality of system-generated answers to disease-specific frequently asked questions (FAQs) in four CTDs: Systemic lupus erythematosus (SLE), idiopathic inflammatory myopathy (IIM), Sjögren disease (SjD), and systemic sclerosis (SSc). Reporting was reviewed against the STROBE guideline for observational studies and the GAMER Statement for studies involving generative artificial intelligence tools in medical research. Completed reporting checklists are provided as supplementary files [25, 26]. The four CTDs were selected a priori because of their prevalence, dominant organ manifestations, treatment complexity, prognosis and high patient needs, thereby allowing assessment across a clinically heterogeneous CTD spectrum. In an initial step, we compiled a set of disease-specific FAQ in collaboration with the German patient advocacy organizations for each disease^1^. Specifically, the advocacy organizations were contacted and asked to compile a list of the 20 most important patient questions for their respective disease. Within each organization, questions were curated through an internal consensus process, and a structured selection step through an internal consensus process based on perceived relevance, frequency, and importance for patient education was used to identify the 20 most frequent and highest-priority items. As an additional validation step, the research team reviewed all submitted questions and, where necessary, applied minor linguistic edits to improve clarity and consistency while preserving the original meaning and content.

Three general-purpose LLMs – Claude 4.0 Sonnet (Anthropic, San Francisco, CA, USA), ChatGPT-5 (OpenAI, San Francisco, CA, USA), and Gemini 2.5 Pro (Google LLC, Alphabet Inc., Mountain View, CA, USA) – were selected based on prior diagnostic performance [27, 28]. Given the daily prompt restrictions associated with the free-access subscription, the evaluations were conducted in sequential testing sessions distributed across the established study timeframe conducted between Aug 20 and Oct 07, 2025; each disease was completed sequentially. Moreover, for each disease, we ran Google Search (https://www.google.com/) on a separate day and captured the Google AI Overview response shown for the respective query. All questions were submitted by one user, each question was submitted in a new session without prior context, responses were recorded, and consolidated answer sets were created for subsequent rating by patients and physicians. Raters were blinded to system identity. As LLM outputs and Google Search results are nondeterministic and may change by model version, account settings, region, time, or safety filters, each response should be interpreted as a single time-stamped realization; repeated sampling was not performed.

Adult patients (≥ 18 years) with a confirmed diagnosis of SLE, IIM, SjD, or SSc, as defined by current European League Against Rheumatism/American College of Rheumatology (EULAR/ACR) classification criteria were included and recruited at the University Medical Center Hamburg-Eppendorf. For each disease entity, five patients evaluated the model responses. Health literacy was assessed using the eHealth Literacy Scale (eHEALS) [29] and the cutoff for high eHealth literacy was set > 26 [30]. In addition, a panel of five board-certified rheumatologists independently rated the model outputs. Raters did not receive any rating education before rating the answers. Interrater reliability (IRR) among patient and physician raters was quantified using two-way random-effects, absolute-agreement intraclass correlation coefficients ICCs(2,*k*), reporting ICC(2,1) for single-rater reliability and ICC(2,5) for the mean of five raters.

Standardized evaluation forms were used for both patients and physicians. For each question, raters provided (i) an overall impression as a forced rank order across the four systems (I–IV; I = best, IV = worst) and (ii) domain-specific Likert ratings (1–5; 1 = excellent, 5 = inadequate). Patients rated empathy, comprehensibility, and trustworthiness. Physicians rated medical correctness and additionally provided ratings for empathy and comprehensibility. Lower scores indicated better performance across all Likert outcomes [31, 32]. If a system failed to provide an answer for a given question, no Likert rating was assigned for that item and the overall impression rank was coded as worst for that system. Response rates (proportion of questions answered) were calculated per system.

The primary outcome was the overall impression of the system-generated answers per question, assessed as a forced rank order across the four systems. Secondary outcomes were domain ratings (patients: empathy, comprehensibility, trustworthiness; physicians: medical correctness), which were analyzed as supportive endpoints to characterize specific response dimensions.

For each disease entity and system, outcomes were summarized descriptively as mean ± standard deviation (SD). Differences in ratings across the four systems were assessed using Friedman’s test for related samples; if the overall test was significant, pairwise post hoc comparisons were performed using Dunn’s multiple-comparisons procedure with adjusted p values. For domain-specific analyses, only items for which all four systems provided an answer were included.

To explore potential differences between question categories, predefined content clusters (A–E, see Results for definition) were compared using a repeated-measures mixed-effects model with Greenhouse-Geisser correction and Tukey-adjusted post hoc tests. Normally distributed continuous variables were summarized as mean ± standard deviation, whereas non-normally distributed variables were summarized as median (Q1–Q3). Categorical variables were reported as counts and percentages. Given the small sample size and exploratory design, statistical analyses were interpreted descriptively, and all analyses were exploratory.

Using a structured guideline-coverage mapping, we assessed the extent to which contemporary EULAR guidelines (where available) address the 20 pre-specified patient questions per disease. EULAR guidelines were selected as these reflect the primary local guideline reference for this study [4, 5, 33, 34]. Each question was scored as 0 (not addressed), 1 (partially addressed), or 2 (explicitly addressed). Initial ratings were performed independently by two raters, disagreements were resolved by consensus, and interrater agreement was quantified with linear weighted Cohen’s kappa. Results were summarized descriptively overall and by disease.

Statistical significance was set at *p* < 0.05. All statistical analyses were performed using Microsoft Excel (Microsoft Corporation, Redmond, WA, USA), Prism software (version 10, GraphPad Software, La Jolla, CA, USA) and R software (R Foundation for Statistical Computing).

The study was conducted in accordance with the Declaration of Helsinki and approved by the local ethics committee of the Medical Association Hamburg, Hamburg, Germany (protocol number: 2025-101618-BO-ff; approval date: 16th December, 2025). All participants provided informed consent.

## Results

### Consensus-based question sets

Twenty disease-specific FAQs per disease were curated (total *n* = 80), see supplementary 1.

### Response completeness for evaluated systems

ChatGPT-5, Gemini 2.5 Pro, and Claude 4.0 Sonnet generated answers for all questions (80/80, response rate 100%), whereas Google Search generated answers for 90% of questions (72/80).

### Patient ratings

Patient characteristics are presented in Table 1. 60% of patients were female, with a median age of 53 years (IQR 29) and a median disease duration of 55 months (IQR 70). Most patients exhibited high health literacy (eHEALS ≥ 26; 85%), with a mean eHEALS score of 29.05 (SD 3.71).

**Fig. 1:**
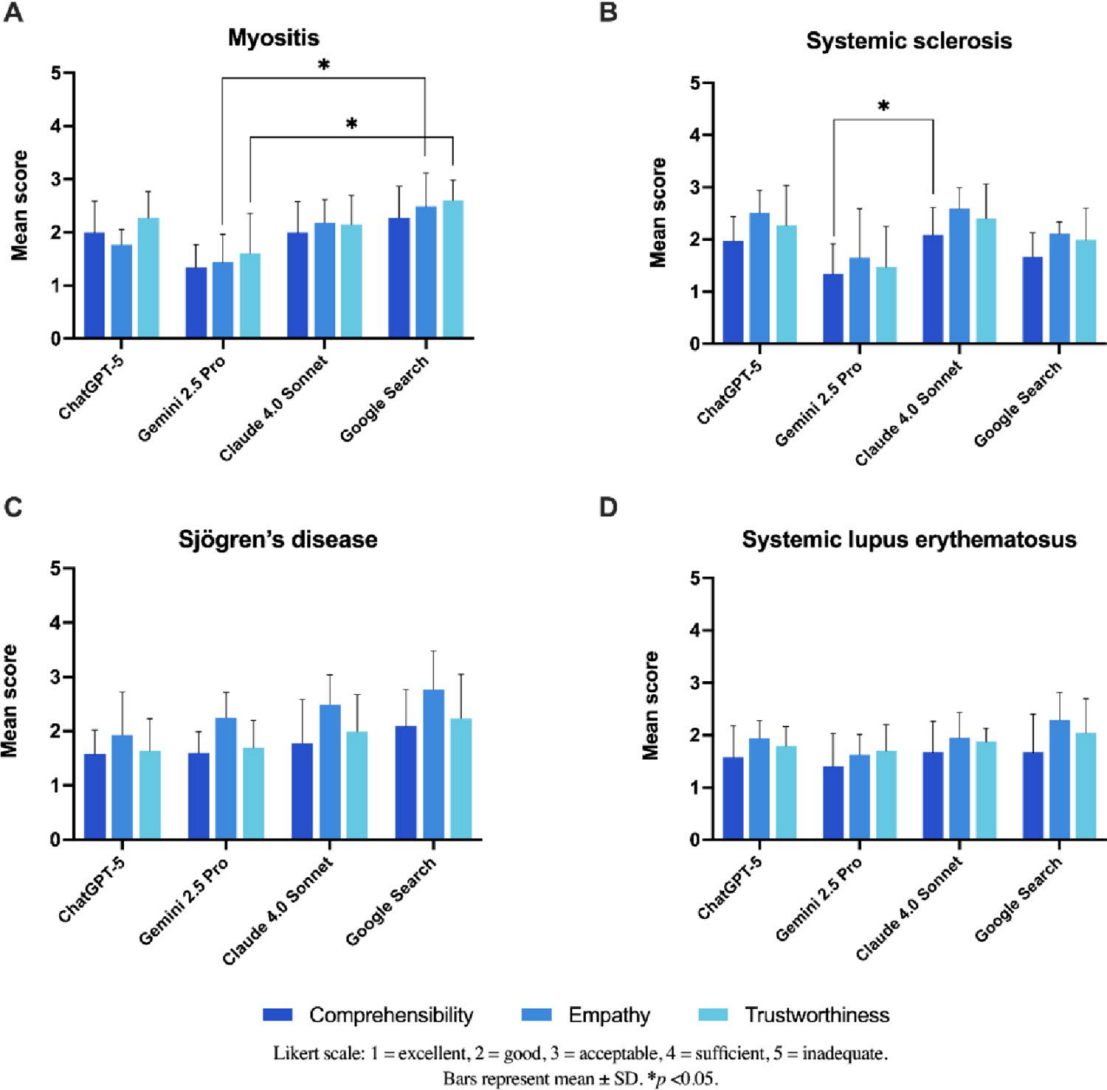
Mean patient rating scores for responses concerning four connective tissue diseases generated by ChatGPT-5, Gemini 2.5 Pro, Claude 4.0 Sonnet, and Google Search. For each disease, outputs were evaluated on four domains - comprehensibility (dark blue), empathy (blue and trustworthiness (light blue) - using a 5-point Likert-type scale (lower scores indicate better ratings). Bars show mean scores and error bars indicate standard deviation across ratings. Statistically significant pairwise differences between systems are indicated by brackets (*p* < 0.05). **A** Idiopathic inflammatory myopathy, **B** Systemic sclerosis, **C** Sjögren disease, and **D** systemic lupus erythematosus. 20 questions per disease; complete-case items only

Overall, patients rated Gemini 2.5 Pro most favorably across diseases and domains (empathy, comprehensibility, trustworthiness, and overall impression), whereas Google Search was most frequently rated least favorably in 68.8% (*n* = 11/16) (Figs. 1 and 3A).

Interrater reliability was low for single ratings (ICC (2,1) = 0.068–0.204; 95% CI 0.017–0.267) and improved to low-to-moderate for mean of five ratings (ICC (2,5) = 0.268–0.562; 95% CI 0.079–0.646).

Across systems and diseases, patients provided generally favorable ratings for empathy and trustworthiness (mean ratings 1.44–2.76 and 1.47–2.27, respectively; lower scores indicate better performance, Fig. 1). Between-model differences were limited. In IIM, overall differences were detected for empathy, trustworthiness, and overall rating, driven by a single pairwise contrast between Gemini 2.5 Pro and Google Search (supplementary 1, Table 1). In SSc, overall differences emerged for trustworthiness and overall rating, with one significant pairwise contrast between Gemini 2.5 Pro and Claude 4.0 Sonnet (supplementary 1, Table 1). No significant between-model differences were observed in SLE, and in SjD only overall rating differed significantly between ChatGPT-5 and Google Search (supplementary 2, Table 1).

Despite the limited number of statistically significant comparisons, the rank-based overall impression showed a consistent preference pattern: patients most frequently ranked Gemini 2.5 Pro first (59% best; 9% fourth best), whereas Google Search was most often ranked last (55% fourth; 7% best) (Fig. 4A).

### Physician ratings

**Fig. 2:**
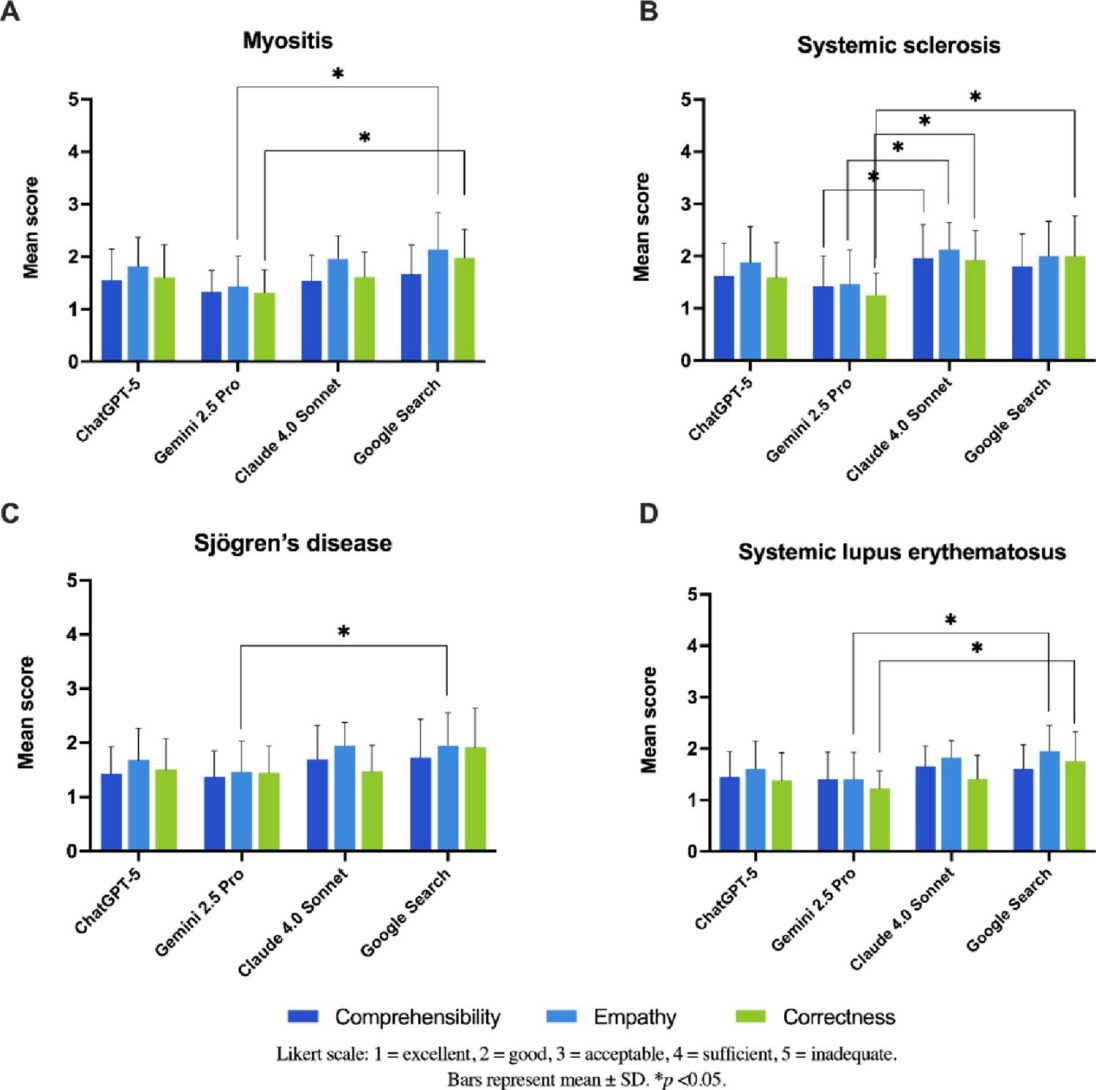
Mean physician rating scores for responses concerning four connective tissue diseases generated by ChatGPT-5, Gemini 2.5 Pro, Claude 4.0 Sonnet, and Google Search. For each disease, outputs were evaluated on four domains—comprehensibility (dark blue), empathy (blue) and correctness (green)—using a 5-point Likert-type scale (lower scores indicate better ratings). Bars show mean scores and error bars indicate standard deviation across ratings. Statistically significant pairwise differences between systems are indicated by brackets (*p* < 0.05). **A** Idiopathic inflammatory myopathy, **B** Systemic sclerosis, **C** Sjögren disease, and **D** systemic lupus erythematosus. 20 questions per disease; complete-case items only

**Fig. 3:**
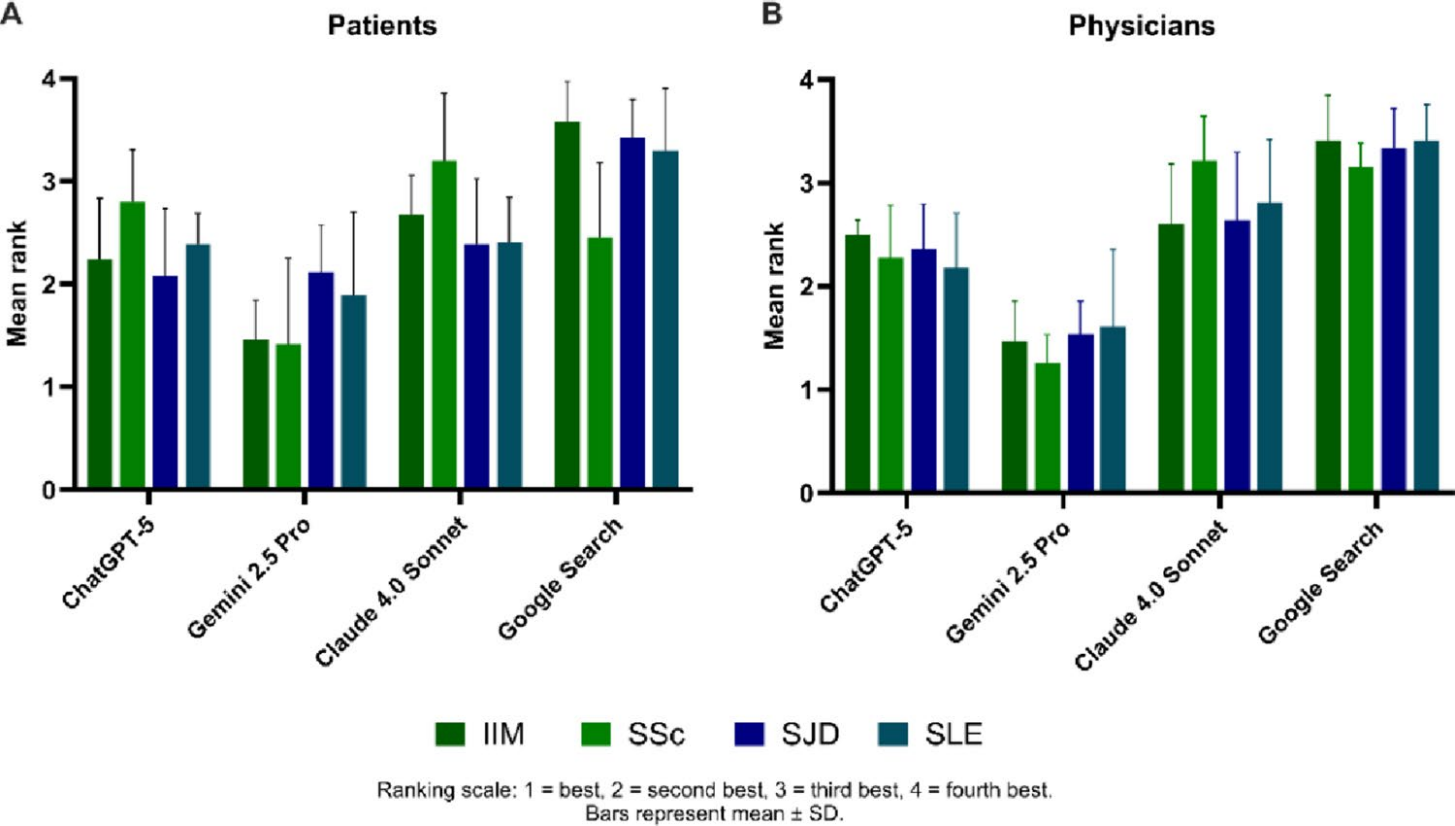
Mean patient and physicians overall rating scores for responses concerning four connective tissue diseases generated by ChatGPT-5, Gemini 2.5 Pro, Claude 4.0 Sonnet, and Google Search. For each disease, outputs were evaluated using a ranking scale; 1 = best, 2 = second best, 3 = third best, 4 = fourth best. Lower scores indicate better overall performance. Bars show mean scores and error bars indicate standard deviation across ratings. **A** Patients, **B** physicians. 20 questions per disease

Physician ratings showed similar patterns across diseases, with Gemini 2.5 Pro achieving the most favorable overall scores (Figs. 2 and 3B). Interrater reliability among the five clinicians was low for single ratings (ICC(2,1) = 0.109; 95% CI 0.091–0.129) and moderate for mean ratings (ICC(2,5) = 0.379; 95% CI 0.332–0.425).

**Fig. 4:**
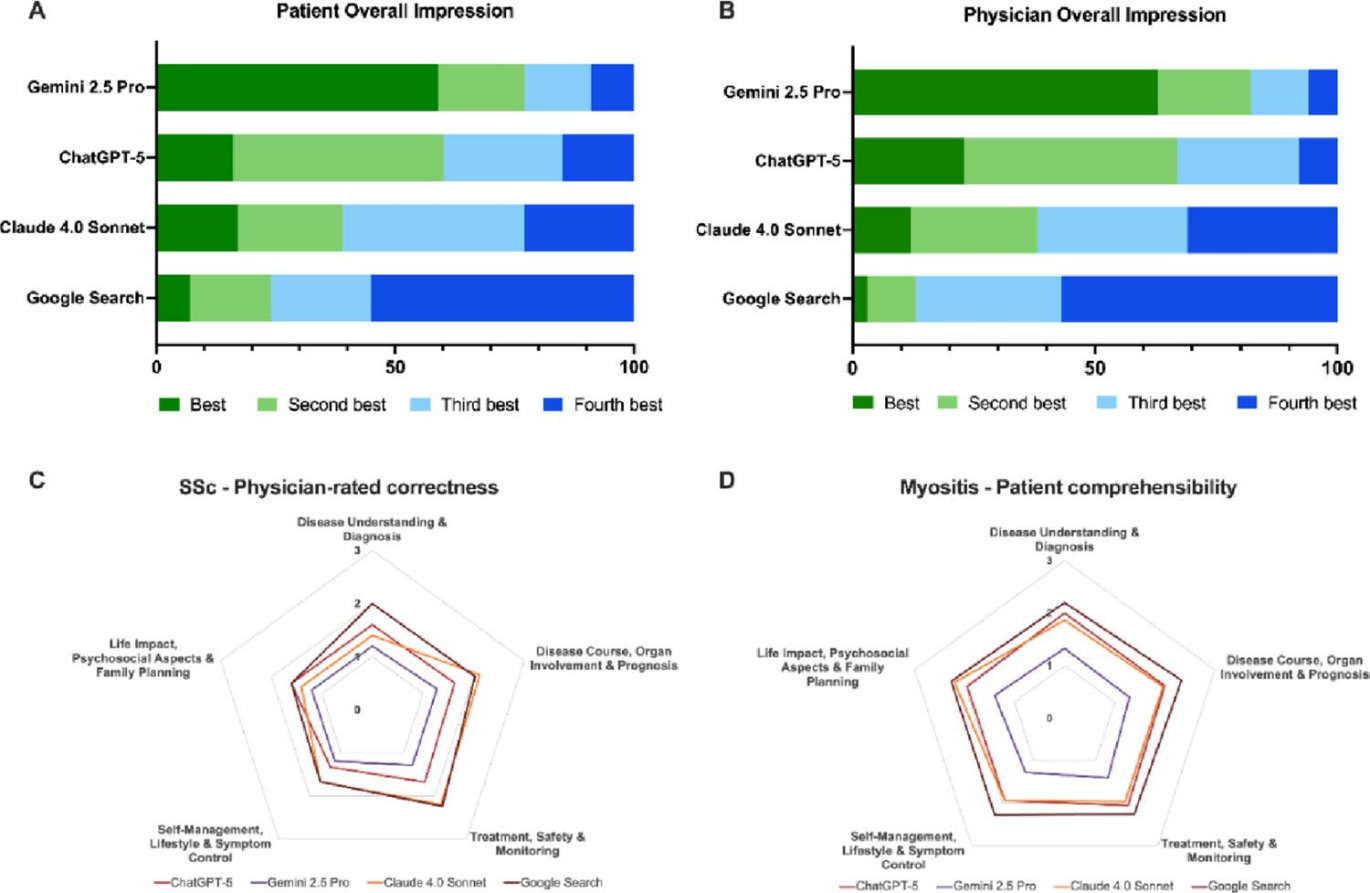
Preference rankings and domain-specific performance profiles of information generated by ChatGPT-5, Gemini 2.5 Pro, Claude 4.0 Sonnet, and Google Search. **A** Patient overall impression: stacked horizontal bars depict the proportion (%) of evaluations in which each system was ranked best, second best, third best, or fourth best. **B** Physician overall impression: analogous ranking distribution by physicians. **C** Systemic sclerosis (SSc) - physician-rated correctness: radar plot of mean scores across five content clusters (disease understanding & diagnosis; disease course, organ involvement & prognosis; treatment, safety & monitoring; self-management, lifestyle & symptom control; life impact, psychosocial aspects & family planning), with larger polygons indicating worse correctness. **D** Myositis-patient-rated comprehensibility (representative example): radar plot of mean comprehensibility scores across the same five domains, with larger polygons indicating worse comprehensibility. Myositis is shown as a representative condition because no significant differences were observed across diseases and outcomes overall

Physician-assessed medical correctness was rated favorably across disease groups and systems (mean ratings 1.23–1.98, Fig. 2). Across the four disease entities, Friedman tests indicated significant between-model differences in empathy, comprehensibility, and overall rating. Dunn-adjusted post hoc comparisons showed that significant pairwise effects predominantly involved Gemini 2.5 Pro, most commonly versus Google Search. Additional significant contrasts were observed versus Claude 4.0 Sonnet in SSc (supplementary 2, Table 2). Consistent with these findings, in rank-based overall impression, physicians most frequently ranked Gemini 2.5 Pro first (63% best; 6% fourth best), whereas Google Search was most often ranked last (57% fourth; 3% best, Fig. 4B).

Patient-physician preference overlap was directionally consistent. Both groups most frequently ranked Gemini 2.5 Pro as the best overall system and Google Search as the least favorable system, suggesting convergence between patient-perceived communication quality and physician global appraisal.

### Exploratory analysis by question content category

Next, FAQs were grouped into five predefined content clusters: *disease understanding and diagnosis* (Cluster A), *disease course*,* organ involvement and prognosis* (Cluster B), *treatment*,* safety and monitoring* (Cluster C), *self-management*,* lifestyle and symptom control* (Cluster D), and *life impact*,* psychosocial aspects and family planning* (Cluster E). Exploratory mixed-effects analyses showed no consistent differences between clusters across outcomes. Isolated cluster-specific differences were observed for physician-rated correctness in SSc (Claude 4.0 Sonnet: cluster A vs. C; adjusted *p* = 0.0435; Fig. 4C) and for physician-rated empathy in SjD (ChatGPT-5: cluster A vs. E; adjusted *p* = 0.0385). Regarding patient-rated domains no significant differences emerged (*p* > 0.05, Fig. 4D).

### Coverage analysis of FAQs by current EULAR guidelines

Finally, coverage of FAQs by current EULAR guidelines was assessed. As no current EULAR guideline for IIM is available, a coverage analysis for IIM was not undertaken. The interrater agreement, using Cohen κ, was high (κ_w_ = 0.867).

**Fig. 5:**
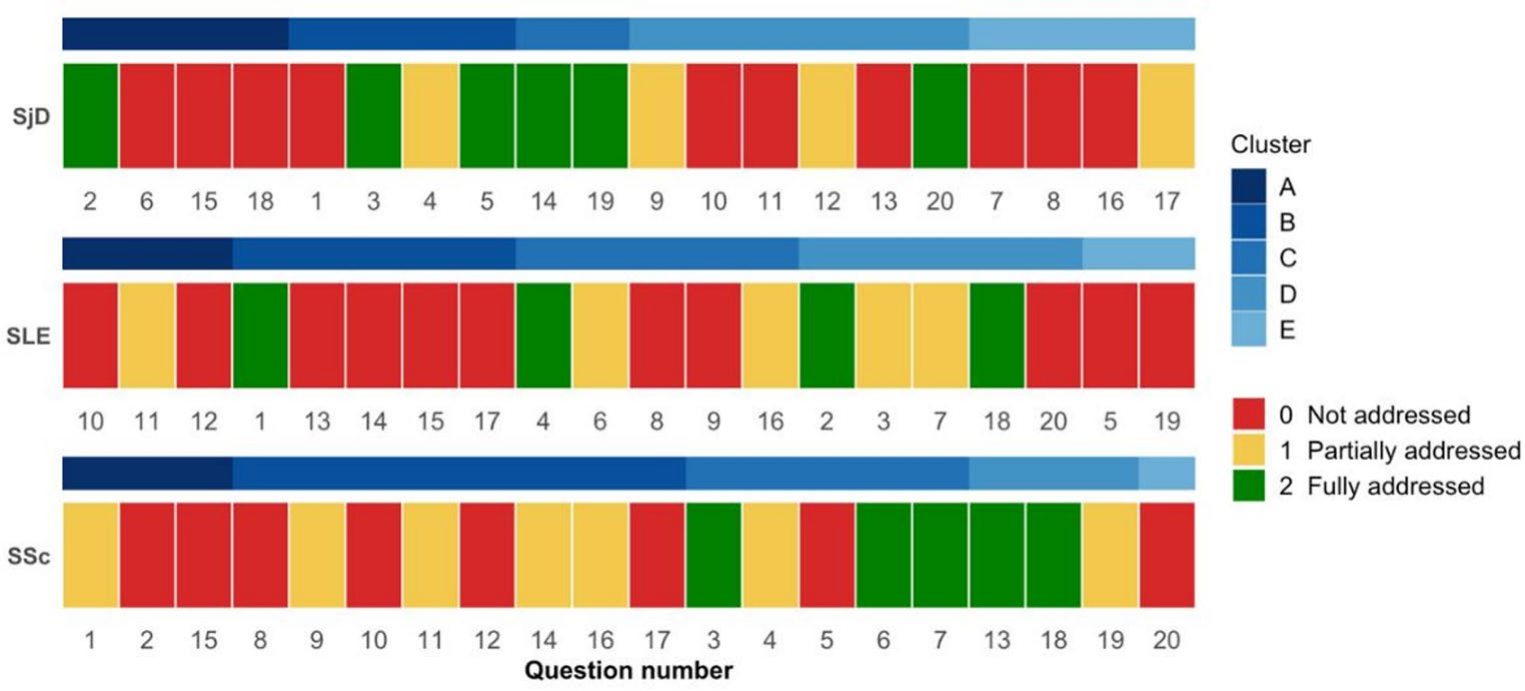
Heatmap of EULAR guideline coverage of patient FAQs across three CTDs. Rows indicate disease entities (Sjögren disease, Systemic lupus erythematosus, Systemic sclerosis) and columns represent individual frequently asked questions (Q1–Q20). Cell colors encode the degree to which each question is addressed by current EULAR recommendations: red = not answered (0), yellow = partially answered (1), and green = fully answered (2). Cluster data values are visualized using a blue-scale gradient: Cluster A, disease understanding and diagnosis = darkest blue, Cluster B, disease course, organ involvement and prognosis) = dark blue, Cluster C, treatment, safety and monitoring = medium blue, Cluster D, self-management, lifestyle and symptom control = light blue, Cluster E, life impact, psychosocial aspects and family planning = lightest blue

Across the three disease areas, only a minority of questions were fully addressed by the current EULAR guidelines. For SjD, 6/20 questions (30%) were fully answered, 4/20 (20%) partially answered, and 10/20 (50%) not answered (Fig. 5). Similarly, in SLE, 4/20 (20%) were fully answered, 5/20 (25%) partially answered, and 11/20 (55%) not answered (Fig. 5). For SSc, 5/20 questions (25%) were fully answered, 7/20 (35%) partially answered, and 8/20 (40%) not answered (Fig. 5). Overall, the proportion of unanswered questions ranged from 40% to 55% across conditions, highlighting substantial evidence gaps despite partial coverage in a subset of items. Across the five predefined clusters, clear differences in coverage emerged. Cluster E (*life impact*,* psychosocial aspects and family planning)* showed the lowest coverage: most items were not answered (score 0: SjD 75%, SLE 100%, SSc 100%) and none were fully addressed (score 2: 0% in all three groups) (Fig. 5). Similarly, Cluster A (*disease understanding and diagnosis*) consistently demonstrated low coverage, with a high proportion of unanswered items (score 0: SjD 75%, SLE 66.7%, SSc 66.7%) and little to no full coverage (score 2: SjD 25%, SLE 0%, SSc 0%).

## Discussion

In this prospective evaluation across four multisystem rheumatic diseases, our aim was to determine whether classic Google search and contemporary freely available LLMs (Claude 4.0 Sonnet, ChatGPT-5 and Gemini 2.5 Pro) can reliably address patient-prioritised information needs. To evaluate this approach, we co-developed disease-specific patient FAQs with advocacy groups and assessed the information delivered by different systems from both patient and physician perspectives. Furthermore, we performed a guideline mapping to identify potential gaps between EULAR guideline coverage and patients FAQs. The study therefore complements work on AI attitudes and diagnostic use by focusing on patient education, stakeholder preference, and guideline-coverage mismatch [15, 19, 21, 28].

First, we were able to show that patients and physicians rated outputs for both LLMs and the Google Search consistently good for empathy (mean 1.40–2.77), trustworthiness (mean 1.47–2.60), and comprehensibility (mean 1.33–2.27, Figs. 1 and 2). These findings align with other cross-specialty evaluations reporting that LLMs are often preferred for patient-facing questions and perform well on communication-related outcomes (e.g., satisfaction, empathy, readability) [18, 35–38]. In rheumatology, comparative studies similarly suggest that patients may favor LLM responses even when specialist-rated accuracy is comparable, indicating that information “delivery” (structure, tone, accessibility) is a key differentiator [24, 39]. However, although patients and physicians rated LLMs empathic and comprehensible, it is noteworthy that fluent answer presentation can inflate perceived credibility [16, 40], underscoring the need for evaluation of medical correctness.

Importantly, physician-rated medical correctness was reassuring overall (mean 1.23–1.98 across models, see Fig. 2), aligning with previous studies, which generally reported good accuracy for patient-education queries [23, 24, 39]. It is important to point out, that in our study answers by LLMs and Google Search were overall medically correct. This finding suggests that, under the study conditions tested, all evaluated systems may be considered potentially safe tools. Accordingly, the common skepticism toward online-sourced health information (“Dr. Google”) [16] may be less justified than commonly assumed, given the increasing quality, accessibility, and consistency of information provided by modern search engines and LLM-based systems. Consequently, LLMs can meaningfully support CTD patient education in routine contexts and explanatory tasks. They should be implemented as adjunctive tools with evidence anchoring (e.g., retrieval to guidelines) and explicit uncertainty signaling to mitigate potential hallucinations [13, 16]. This stance is reinforced by emerging frameworks to quantify LLM fidelity and hallucination risk in clinical tasks [41].

In the overall comparison LLM-generated answers received consistently favorable overall ratings, with Gemini 2.5 Pro showing the strongest aggregate performance across diseases (ranked best in 59% and 63% of answers by patients and physicians, respectively, Fig. 3) outperforming the Google Search (ranked best in only 7% and 3% of answers by patients and physicians, respectively, Fig. 3). Interestingly, Google Search provided no direct answer for 10% of questions, whereas LLMs produced responses for all questions. This could be seen as a disadvantage when patient-critical questions remain unanswered. However, this apparent advantage of LLMs may also represent a limitation, as they tend to generate responses even when reliable information is unavailable, increasing the risk of plausible-sounding but inaccurate answers [42]. It is important to point out that topic- and model-specific variability affects reliability, comprehensiveness, and readability, underscoring that perceived “overall correctness” can mask clinically relevant outliers [43, 44], if medical-correctness is not assessed at the same time. In addition, as we did not repeat prompts, our results cannot quantify response variability across repeated runs or future model versions.

In a secondary analysis, perceived answer quality did not differ significantly across patient-defined clusters (except for physician-rated correctness in SSc [Claude 4.0 Sonnet: cluster A vs. C; adjusted *p* = 0.0435] and for physician-rated empathy in SjD [ChatGPT-5: cluster A vs. E; adjusted *p* = 0.0385] Fig. 4) regarding empathy, comprehensibility, trustworthiness and medical correctness, suggesting broadly stable user-perceived performance across CTD question types. This observation aligns with a recent study comparing ChatGPT-4 to physicians-provided answers, where theme-based stratification of accuracy and completeness likewise showed no significant differences [24]. Thus, the absence of meaningful differences across themes supports scalability across heterogeneous informational intents. However, Forte et al. also analysed overall response choices (ChatGPT-4 vs. human) by theme revealing significant differences in favor of ChatGPT-4 in different domains (e.g. prognosis and comorbidities and therapy efficacy/strategy), which might be explained by higher readability of ChatGPT-4 answers [24]. In our study no comparison to physician’s answers was obtained. Accordingly, future studies should pair thematic stratification with objective safety and validity audits (e.g., guideline concordance, clinically relevant omission detection) to distinguish communication quality from content accuracy [41].

Finally, guideline mapping indicated a structural information-needs gap: 40–55% of patient-prioritised FAQs were not addressed by current EULAR recommendations. The largest deficits were observed in domains such as life impact, psychosocial concerns, and family planning, where 75–100% were not answered (score 0: SjD 75%, SLE 100%, SSc 100%) and none (0%) were fully addressed (see Fig. 5). Notably, for IIM, no current EULAR guideline is available, precluding a comparable coverage assessment. This gap mirrors empirical patient survey data showing widespread unmet information needs among people with rheumatic diseases [45]. However, this mismatch should not be interpreted as a shortcoming of international recommendations, which primarily target evidence-based clinical management rather than comprehensive patient education. Instead, it likely reflects broader, well-described care and implementation gaps, underscoring that guideline publication alone does not fully translate into coverage of patient-perceived needs [46]. Nonetheless, the gap is clinically meaningful in CTDs, where disease complexity and uncertainty are high and patients commonly seek supplementary information online [3, 11, 13]. In this light, LLMs may offer a scalable route to address patient-framed questions in accessible language, but only if positioned as an adjunct to clinician care and evidence-based resources (e.g., guideline-linked retrieval) rather than a substitute [47].

Given the rapid evolution of LLMs [16, 40], the disease-specific FAQ set (see supplementary 2) we developed can be used as a practical benchmark to repeatedly reassess longitudinal performance. It enables repeated evaluation over time and supports the monitoring of governance and safety enhancements, including the implementation of source citation requirements and guardrails to ensure alignment with established clinical guidelines. Beyond this, our FAQ set could serve as a patient-derived reference to inform the development and updating of clinical guidelines, helping align guideline priorities with patient preferences and current unmet information needs.

Despite these positive results, however, there are already recent data suggesting that a cautious, context-sensitive use of LLMs in medical settings is advisable. The present study used German-language, overarching disease-specific questions rather than individualized case scenarios, medication decisions, or urgent safety problems. Language may affect LLM outputs through differences in training data, translation quality, terminology, cultural framing, health-system context, and availability of Google Search. For instance, recent data show that even if LLMs do well on medical tests, using them in real life does not necessarily help people make better decisions and can sometimes make decisions worse [48]. This is partly because user–LLM interactions can introduce errors that standard benchmarks do not capture. Moreover, LLMs remain susceptible to adopting and reproducing false recommendations, particularly when misinformation is embedded in realistic clinical documents [49].

Several limitations also warrant consideration. This single-centre study used German-language FAQs from four CTDs, limiting generalisability across other RMDs. We evaluated freely available, general-purpose tools; performance may differ for specialized medical models or retrieval-augmented systems. Outcomes were perception-based and did not assess downstream endpoints such as knowledge gain, behaviour change, or HL improvement - areas with established relevance for patient decision support [50]. The patient sample was limited in size and largely comprised individuals with moderate to high eHealth and health literacy. This may have influenced perceptions of comprehensibility, trustworthiness, and safe use, and may limit the generalizability of our findings to populations with lower health literacy or restricted digital access. Similarly, the physician panel was relatively small, and single-rater reliability was limited, underscoring the need for cautious interpretation of individual ratings. Taken together, these aspects suggest that our findings should be regarded as exploratory and hypothesis-generating, providing a basis for further validation in larger and more diverse cohorts.

## Conclusion

In summary, contemporary general-purpose LLMs produced CTD FAQ responses that were perceived as highly empathic as well as trustworthy and were rated as medically correct by rheumatologists in this blinded evaluation. At the same time, the substantial gap between patient-prioritized questions and guideline coverage highlights the opportunity to develop patient-centered, evidence-grounded information resources for patients. With appropriate safeguards, transparency, and periodic re-evaluation, LLMs might support patient education as a valuable tool.

**Table 1:** Patient characteristics

Characteristic	Group	Total *n* (%)
Gender	Female	12 (60)
	Male	8 (40)
Age, years	18–39	7 (35)
	40–59	7 (35)
	> 60	6 (30)
	Median (Q1-Q3)	53 (34–63)
Disease duration, months	< 12	2 (10)
	12–59	9 (45)
	> 60	9 (45)
	Median (Q1-Q3)	55 (18–88)
Education	No school-leaving certificate	0 (0)
	Basic secondary school certificate	4 (20)
	Secondary school certificate	7 (35)
	German A-level equivalent	2 (10)
	Completed university degree	7 (35)
eHealth literacy, *eHEALS score (points)*	Low (< 20)	0 (0)
	Middle (21–26)	3 (15)
	High (> 27)	17 (85)
	Mean (SD)	29.05 (3.71)

Participant characteristics of the study sample (*N* = 20), including sociodemographic variables, disease duration, educational level, and eHealth literacy

## Electronic Supplementary Material

Below is the link to the electronic supplementary material.

Supplementary file 1

Supplementary file 2

Supplementary file 3

Supplementary file 4

## Footnotes

1 Lupus Erythematosus Selbsthilfegemeinschaft e.V., Sjögren-Selbsthilfe Leipzig, Sklerodermie Selbsthilfe e.V., Myositis Gruppe – Deutsche Gesellschaft für Muskelkranke e.V., and the Dutch patient association for neuromuscular diseases

## Declarations

### Disclaimer

No part of this report has been copied or published elsewhere in whole or in part, except for any related congress abstract explicitly disclosed above. The submitted manuscript is original and is not under consideration elsewhere.
