# Supplementary material for: A patient-derived benchmark for evaluating large language models in connective tissue diseases: blinded multi-stakeholder assessment and guideline comparison
Source: Rheumatol Int. 2026 Jul 14;46(8):210. doi: 10.1007/s00296-026-06178-1 (PMC13364936; doi:10.1007/s00296-026-06178-1)
Supplement: Supplementary file 1 [file 296_2026_6178_MOESM1_ESM.docx]

**Supplementary data 1:**

**Overview of all patient questions**

**Sjögren disease**

1. How does Sjögren disease affect life expectancy?
2. What are the most common Sjögren disease symptoms?
3. How does Sjögren disease affect internal organs?
4. Does Sjögren disease have flares?
5. Can Sjögren disease affect the nervous system?
6. What does “connective tissue disease” mean in Sjögren disease?
7. What legal or workplace support is available for Sjögren disease?
8. What challenges exist in combining employment and Sjögren disease?
9. Which self-help measures can relieve Sjögren disease symptoms?
10. How does diet affect Sjögren disease?
11. Which physical activities are suitable with Sjögren disease?
12. What helps with swollen salivary glands in Sjögren disease?
13. How can I support my immune system with Sjögren disease?
14. Are there current clinical trials for Sjögren disease?
15. Is Sjögren disease genetic or acquired?
16. Can Sjögren disease be passed to children?
17. How can a parent’s Sjögren disease affect a child’s health?
18. When is a salivary gland or lip biopsy indicated for Sjögren disease?
19. Which medications are used to treat Sjögren disease?
20. How is fatigue treated in Sjögren disease?

**Systemic lupus erythematosus**

1. Which organs should be monitored in SLE?
2. Why is sun protection important in SLE?
3. What should I do during an SLE flare?
4. Can I stop SLE medication when I feel better?
5. Can I become pregnant while on SLE medication?
6. What are the benefits and risks of cortisone in SLE?
7. Can diet influence SLE disease course?
8. Which blood tests reflect SLE activity?
9. Why can SLE symptoms flare despite normal blood tests?
10. Why is SLE more common in women?
11. What triggers SLE?
12. Why is SLE diagnosis often delayed?
13. How does SLE affect life expectancy?
14. Can SLE be cured?
15. How likely is dialysis in SLE?
16. How does hydroxychloroquine work in SLE?
17. Can I develop another rheumatic disease in addition to SLE?
18. What should I pay special attention to with SLE?
19. Is SLE hereditary?
20. Is it safe to drink a glass of alcohol with SLE?

**Systemic sclerosis**

1. What types of systemic sclerosis exist?
2. What causes systemic sclerosis?
3. Which treatments are available for systemic sclerosis?
4. What side effects can systemic sclerosis medications cause?
5. Can systemic sclerosis medications harm organs?
6. Which new systemic sclerosis therapies are being studied?
7. What are alternatives to MTX and immunosuppressants in systemic sclerosis?
8. Is a cure for systemic sclerosis expected?
9. What is the typical course of systemic sclerosis?
10. Can systemic sclerosis be stopped or reversed?
11. How do I know if systemic sclerosis affects my organs?
12. How do I recognize a systemic sclerosis flare?
13. Which treatments help Raynaud’s and skin symptoms in systemic sclerosis?
14. How might my systemic sclerosis progress?
15. Why was the term CREST changed?
16. How can organ involvement be detected early in systemic sclerosis?
17. How does systemic sclerosis progress with age?
18. Which rehabilitation, physiotherapy, and occupational therapy services are covered by health insurance for systemic sclerosis?
19. How can I prevent complications from systemic sclerosis?
20. How long can I keep working with systemic sclerosis?

**Idiopathic inflammatory myopathy**

1. What are the main types of myositis?
2. What is the typical course of myositis?
3. What happens in the body in myositis?
4. Is myositis a chronic disease?
5. How do myositis medications work?
6. How can I reduce side effects from myositis medications?
7. How likely is wheelchair dependence in myositis?
8. Can I continue working with myositis?
9. Can I become pregnant with myositis?
10. Is myositis hereditary?
11. Which organs can be affected in myositis?
12. What can I do for myself to relieve myositis symptoms?
13. How can I connect/exchange with others who also suffer from myositis?
14. Which follow-up appointments are needed in myositis?
15. What non-immunosuppressive treatments exist for myositis?
16. Which sports are safe with myositis?
17. Can diet help improve myositis?
18. Is fatigue a symptom of myositis?
19. Is there an increased cancer risk in myositis?
20. What do myositis-specific autoantibodies indicate?
