# Supplementary material for: A patient-derived benchmark for evaluating large language models in connective tissue diseases: blinded multi-stakeholder assessment and guideline comparison
Source: Rheumatol Int. 2026 Jul 14;46(8):210. doi: 10.1007/s00296-026-06178-1 (PMC13364936; doi:10.1007/s00296-026-06178-1)
Supplement: Supplementary file 2 [file 296_2026_6178_MOESM2_ESM.docx]

**Supplemental data 2:**

**Supplementary Table 1: Significant pairwise differences between models (Dunn’s post hoc test, adjusted p-values) in patients rating by disease entity and outcome.** *All pairwise comparisons not listed were not significant (p_adj ≥ 0.05).*

| Disease | Outcome | Significant pairwise comparison | Mean ± SD | Adjusted p-value |
| --- | --- | --- | --- | --- |
| **SSc** | Trustworthiness | **Gemini 2.5 Pro vs Claude 4.0 Sonnet** | 1.47 ± 0.77 vs 2.44 ± 0.88 | 0.0291 |
| **SSc** | Overall rating | **Gemini 2.5 Pro vs Claude 4.0 Sonnet** | 1.42 ± 0.85 vs 3.20 ± 0.89 | 0.0423 |
| **IIM** | Empathy | **Gemini 2.5 Pro vs Google Search** | 1.44 ± 1.02 vs 2.49 ± 0.93 | 0.0087 |
| **IIM** | Trustworthiness | **Gemini 2.5 Pro vs Google Search** | 1.61 ± 0.83 vs 2.60 ± 0.93 | 0.0291 |
| **IIM** | Overall rating | **Gemini 2.5 Pro vs Google Search** | 1.46 ± 0.86 vs 3.60 ± 0.71 | 0.0036 |
| **SjD** | Overall rating | **ChatGPT-5 vs. Google Search** | 2.08 ± 1.06 vs 3.44 ± 0.97 | 0.0423 |

**Supplementary Table 2: Significant pairwise differences between models (Dunn’s post hoc test, adjusted p-values) in physicians rating by disease entity and outcome.** *All pairwise comparisons not listed were not significant (p_adj ≥ 0.05).*

| Disease | Outcome | Significant pairwise comparison | Mean ± SD | Adjusted p-value |
| --- | --- | --- | --- | --- |
| SSc | Comprehensibility | Gemini 2.5 Pro vs Claude 4.0 Sonnet | 1.33 ± 0.59 vs 1.96 ± 0.86 | 0.0291 |
| SSc | Empathy | Gemini 2.5 Pro vs Claude 4.0 Sonnet | 1.46 ± 0.70 vs 2.13 ± 0.68 | 0.0036 |
| SSc | Correctness | Gemini 2.5 Pro vs Claude 4.0 Sonnet | 1.25 ± 0.52 vs 1.93 ± 0.91 | 0.0423 |
| SSc | Correctness | Gemini 2.5 Pro vs Google Search | 1.25 ± 0.52 vs 2.00 ± 1.08 | 0.0087 |
| SSc | Overall rating | Gemini 2.5 Pro vs Google Search | 1.26 ± 0.87 vs 3.24 ± 0.81 | 0.0197 |
| IIM | Empathy | Gemini 2.5 Pro vs Google Search | 1.43 ± 0.69 vs 2.15 ± 0.88 | 0.0423 |
| IIM | Correctness | Gemini 2.5 Pro vs Google Search | 1.32 ± 0.60 vs 1.98 ± 1.00 | 0.0014 |
| IIM | Overall rating | Gemini 2.5 Pro vs Google Search | 1.47 ± 0.88 vs 3.44 ± 0.81 | 0.0087 |
| SjD | Empathy | Gemini 2.5 Pro vs Google Search | 1.46 ± 0.67 vs 1.95 ± 0.97 | 0.0423 |
| SjD | Overall rating | Gemini 2.5 Pro vs Google Search | 1.54 ± 0.82 vs 3.44 ± 0.74 | 0.0057 |
| SLE | Empathy | Gemini 2.5 Pro vs Google Search | 1.40 ± 0.59 vs 1.96 ± 0.90 | 0.0036 |
| SLE | Correctness | Gemini 2.5 Pro vs Google Search | 1.23 ± 0.55 vs 1.76 ± 0.96 | 0.0291 |
| SLE | Overall rating | Gemini 2.5 Pro vs Google Search | 1.61 ± 0.92 vs 3.41 ± 0.79 | 0.0197 |
