# Supplementary material for: A patient-derived benchmark for evaluating large language models in connective tissue diseases: blinded multi-stakeholder assessment and guideline comparison
Source: Rheumatol Int. 2026 Jul 14;46(8):210. doi: 10.1007/s00296-026-06178-1 (PMC13364936; doi:10.1007/s00296-026-06178-1)
Supplement: Supplementary file 3 [file 296_2026_6178_MOESM3_ESM.docx]

**Supplementary File: GAMER Checklist**

Manuscript: A Patient-Derived Benchmark for Evaluating Large Language Models in Connective Tissue Diseases: Blinded Multi-Stakeholder Assessment and Guideline Comparison

| **GAMER item** | **Recommendation** | **Where addressed** |
| --- | --- | --- |
| **General declaration 1** | State whether generative AI tools were used in any part of the study or manuscript. | The manuscript clearly evaluates Claude 4.0 Sonnet, ChatGPT-5, Gemini 2.5 Pro and Google Search/AI Overview as study systems. |
| **GAI tool specifications 2** | Specify each GAI tool, provider, version/release date where available, access mode, and date/period of use. | The manuscript lists Claude 4.0 Sonnet, ChatGPT-5 and Gemini 2.5 Pro with providers and gives the testing period from Aug 20 to Oct 07, 2025. All tools were accessed through the web interface in a free version. |
| **Prompting techniques and unedited outputs 3** | Describe the prompting approach and provide prompts and unedited responses where feasible. | The manuscript states that all questions were submitted by one user, each in a new session without prior context, and that responses were recorded. The full question list is included and the prompt was only the question itself. Outputs were not edited. |
| **New or fine-tuned GAI model 4** | Declare whether a new GAI model was developed or an existing model was fine-tuned. | No new or fine-tuned model is described. The study uses general-purpose, freely available LLMs. |
| **Role of GAI tools in the study 5** | Describe the role of GAI tools in all study phases in which they were used. | The role of LLMs as answer-generating systems for patient-derived questions is described. |
| **AI-assisted manuscript sections 6** | Report specific manuscript sections or paragraphs to which GAI tools contributed. | During the preparation of this work the authors used ChatGPT-5 in order to enhance the readability of this work. After using this tool/service, the authors reviewed and edited the content as needed and take full responsibility for the content of the publication. |
| **Content verification 7** | Explain how AI-generated content was checked and, when necessary, modified. | The study includes blinded ratings by patients and rheumatologists, physician assessment of medical correctness, and guideline coverage mapping. Model outputs were not edited before rating and only anonymized/formatted. |
| **Data privacy and confidentiality 8** | Describe how privacy and confidentiality were protected during GAI use. | Ethics approval and informed consent are reported. No patient-identifiable information, individual clinical records, or rating data were submitted to any GAI tool. |
| **Impact on interpretation and conclusions 9** | Discuss whether and how GAI use may have affected interpretation, accuracy, or conclusions. | The discussion addresses nondeterminism, model/version changes, lack of repeated sampling, hallucination risk, general-purpose tools, and the need for safeguards. |

***Guideline citation:*** *Luo X, Tham YC, Giuffre M, et al. Reporting guideline for the use of Generative Artificial Intelligence tools in Medical Research: the GAMER Statement. BMJ Evidence-Based Medicine. 2025;30(6):390-400. doi:10.1136/bmjebm-2025-113825.*

***Abbreviations:*** *CTD, connective tissue disease; EULAR, European Alliance of Associations for Rheumatology; FAQ, frequently asked question; GAI, generative artificial intelligence; GAMER, Generative Artificial intelligence tools in MEdical Research; LLM, large language model.*
