# Supplementary material for: A patient-derived benchmark for evaluating large language models in connective tissue diseases: blinded multi-stakeholder assessment and guideline comparison
Source: Rheumatol Int. 2026 Jul 14;46(8):210. doi: 10.1007/s00296-026-06178-1 (PMC13364936; doi:10.1007/s00296-026-06178-1)
Supplement: Supplementary file 4 [file 296_2026_6178_MOESM4_ESM.docx]

**Supplementary File: STROBE Checklist**

Manuscript: A Patient-Derived Benchmark for Evaluating Large Language Models in Connective Tissue Diseases: Blinded Multi-Stakeholder Assessment and Guideline Comparison

| **STROBE item** | **Recommendation** | **Where addressed / audit status in revised manuscript** |
| --- | --- | --- |
| **Title/abstract 1a** | Indicate the study design with a commonly used term in the title or abstract. | The Abstract Methods identifies the study as a prospective single-center observational study. |
| **Title/abstract 1b** | Provide an informative and balanced summary of what was done and found. | The Abstract summarizes FAQ development, evaluated systems, patient and physician ratings, response completeness, ranking results, reliability, and guideline coverage. |
| **Background/rationale 2** | Explain the scientific background and rationale for the investigation being reported. | The Introduction describes CTD information needs, health literacy, online health information seeking, and the emerging rheumatology-specific LLM literature. |
| **Objectives 3** | State specific objectives, including any prespecified hypotheses. | The Introduction states three objectives: disease-specific FAQ development, patient/physician evaluation of system-generated answers, and EULAR guideline mapping. No formal hypothesis is stated because the study is exploratory. |
| **Study design 4** | Present key elements of study design early in the paper. | The Methods section opens with the prospective, single-center observational benchmarking design and specifies the four CTDs. |
| **Setting 5** | Describe the setting, locations, and relevant dates, including periods of recruitment, exposure, follow-up, and data collection. | The Methods states the University Medical Center Hamburg-Eppendorf setting and LLM/Google testing timeframe (Aug 20-Oct 07, 2025). Patient recruitment was done at University Medical Center Hamburg-Eppendorf with patients seen in the III. Department of Medicine, Section for Rheumatology either in inpatient or outpatient setting. |
| **Participants 6a** | Give the eligibility criteria, and the sources and methods of selection of participants. | The Methods describes adult patients with confirmed SLE, IIM, Sjogren disease, or systemic sclerosis, recruited at the University Medical Center Hamburg-Eppendorf, and a panel of five board-certified rheumatologists. |
| **Participants 6b** | For matched studies, give matching criteria and number of exposed and unexposed. | Not applicable. This was not a matched observational study. |
| **Variables 7** | Clearly define all outcomes, exposures, predictors, potential confounders, and effect modifiers. | The Methods defines the evaluated systems, the primary forced-rank outcome, secondary Likert domains, response completeness, eHEALS, and EULAR guideline coverage scores. |
| **Data sources/measurement 8** | For each variable of interest, give sources of data and details of assessment methods. Describe comparability of assessment methods if there is more than one group. | The Methods describes advocacy-derived FAQ generation, single-session question submission, response recording, blinded rating forms, eHEALS assessment, and independent EULAR guideline mapping with consensus resolution. |
| **Bias 9** | Describe any efforts to address potential sources of bias. | The Methods describes blinded assessment, standardized rating forms, new sessions without prior context, independent guideline mapping, and consensus resolution. The Discussion addresses residual bias from small sample size, high eHealth literacy, language, model variability, and nondeterministic outputs. |
| **Study size 10** | Explain how the study size was arrived at. | The Results/Table 1 report the study size and the Discussion acknowledges the modest sample size in rare CTDs. The Methods should explicitly state that no formal sample-size calculation was performed and that the sample was pragmatic/exploratory, if this sentence is not already present. |
| **Quantitative variables 11** | Explain how quantitative variables were handled in the analyses. If applicable, describe which groupings were chosen and why. | The Methods describes handling of Likert scores, rank orders, eHEALS cutoff categories, response rates, ICCs, and guideline coverage scores (0-2). |
| **Statistical methods 12a** | Describe all statistical methods, including those used to control for confounding. | The Methods reports descriptive summaries, ICCs, Friedman tests, Dunn-adjusted post hoc comparisons, mixed-effects models, Greenhouse-Geisser correction, Tukey adjustment, and weighted kappa for guideline mapping. |
| **Statistical methods 12b** | Describe any methods used to examine subgroups and interactions. | The Methods describes exploratory analyses by predefined question-content clusters and disease entities. |
| **Statistical methods 12c** | Explain how missing data were addressed. | The Methods states that unanswered items did not receive Likert ratings, that the corresponding rank was coded as worst, and that domain-specific analyses used complete-case items in which all systems provided an answer. |
| **Statistical methods 12d** | If applicable, describe analytical methods taking account of sampling strategy. | Not applicable. No complex sampling design or weighting strategy was used. |
| **Statistical methods 12e** | Describe any sensitivity analyses. | Not applicable. No formal sensitivity analyses or repeated sampling were performed. |
| **Participants/results 13a** | Report numbers of individuals at each stage of study. | Results and Table 1 report 20 patients and five physicians. Response completeness is reported per evaluated system. |
| **Participants/results 13b** | Give reasons for non-participation at each stage. | Not applicableThe manuscript reports included participants and evaluators; reasons for non-participation are not central to the benchmarking design but should be added if screening or refusal data were collected. |
| **Participants/results 13c** | Consider use of a flow diagram. | Not applicable. A flow diagram is not essential for this small single-center benchmarking study; numbers are reported in text and Table 1. |
| **Descriptive data 14a** | Give characteristics of study participants and information on exposures and potential confounders. | Table 1 reports gender, age, disease duration, education, and eHealth literacy. The Methods describes the evaluated systems and procedures. |
| **Descriptive data 14b** | Indicate number of participants with missing data for each variable of interest. | Google non-responses are explicitly reported. For patient participants no data are missing. |
| **Descriptive data 14c** | For cohort studies, summarize follow-up time. | Not applicable. The study did not include longitudinal clinical follow-up. |
| **Outcome data 15** | Report numbers of outcome events or summary measures. | Results report response rates, mean ratings, rank percentages, ICCs with confidence intervals, and guideline coverage frequencies. |
| **Main results 16a** | Give unadjusted estimates and, if applicable, confounder-adjusted estimates and their precision. | Results emphasize descriptive mean ratings, standard deviations, rank percentages, response rates, ICC estimates with confidence intervals, and exploratory p values. No confounder-adjusted analyses were planned. |
| **Main results 16b** | Report category boundaries when continuous variables were categorized. | The Methods/Table 1 state the eHEALS cutoff categories and age/disease-duration categories. |
| **Main results 16c** | If relevant, translate estimates of relative risk into absolute risk for a meaningful time period. | Not applicable. The study did not estimate relative risks or incidence rates. |
| **Other analyses 17** | Report other analyses done, such as subgroup analyses, interactions, and sensitivity analyses. | Results include exploratory content-cluster analyses and EULAR guideline coverage mapping. No formal sensitivity analyses were performed. |
| **Key results 18** | Summarize key results with reference to study objectives. | The Discussion opening and Conclusion summarize the key findings in relation to FAQ development, stakeholder evaluation, and guideline coverage. |
| **Limitations 19** | Discuss limitations, taking into account sources of potential bias or imprecision. | The Discussion addresses single-center design, German-language questions, high eHealth literacy, small patient and physician samples, low single-rater reliability, nondeterministic outputs, and lack of systematic hallucination auditing. |
| **Interpretation 20** | Give a cautious overall interpretation of results considering objectives, limitations, multiplicity of analyses, related studies, and other relevant evidence. | The Discussion and Conclusion were softened to avoid claims of clinical superiority or safety and to frame findings as exploratory and adjunctive. |
| **Generalisability 21** | Discuss the generalisability/external validity of the study results. | The Discussion addresses generalizability across languages, health-literacy groups, health-system contexts, model versions, other RMDs, and specialized medical/retrieval-augmented systems. |
| **Funding 22** | Give the source of funding and the role of the funders for the present study and, if applicable, for the original study on which the present article is based. | The Declarations section should include funding, role of funders, conflicts of interest, and Open Access funding eligibility. This information must be completed according to the final author/funder information. |

*Abbreviations: CTD, connective tissue disease; eHEALS, eHealth Literacy Scale; EULAR, European Alliance of Associations for Rheumatology; FAQ, frequently asked question; ICC, intraclass correlation coefficient; LLM, large language model; RMD, rheumatic and musculoskeletal disease.*
